# Disproportionately Increased Incidence of Anaplastic Thyroid Cancer in the North Yorkshire Region: A UK Tertiary Centre Study

**DOI:** 10.7759/cureus.72006

**Published:** 2024-10-21

**Authors:** Ayodeji Adedeji, Eugene Omakobia, Charles Ojo, Andrew Coatesworth, Frank Agada

**Affiliations:** 1 Department of Head and Neck Surgery (ENT), York and Scarborough Teaching Hospitals NHS Foundation Trust, York, GBR; 2 Department of Emergency Medicine, Pilgrim Hospital, United Lincolnshire Hospitals NHS Trust, Boston, GBR

**Keywords:** anaplastic, cancer, dysphagia, head and neck surgery, neck lump, north yorkshire, thyroid, united kingdom

## Abstract

Globally, the incidence of anaplastic thyroid cancer (ATC) has followed a stable trend. However, over the past decade, we have observed a marked rise in the number of cases in the North Yorkshire region of the United Kingdom. Our study aimed to explore the incidence, demographics, and geographical distribution of ATC in this region over a 12-year period. We retrieved the clinical records of all patients with ATC in our centre from 2010 to 2022 and determined the incidence, demographics, and geographical spread of the data. 20 patients were diagnosed with ATC within the data collection period, with an average annual incidence of 14 per 10,000,000 of the entire North Yorkshire population (35 per 10,000,000 age-adjusted incidence rate). There was a clustering of data around the North Yorkshire region centre. The incidence of ATC in North Yorkshire was geometrically higher than the national average of 1.7 per 10,000,000. The geographical clustering around the region's centre was likely explained by its higher population density.

## Introduction

Over the last decade, a notable increase in the incidence of thyroid cancers has been reported globally [1]. This is largely due to the more widespread use of advanced imaging techniques as part of the diagnostic work-up for head and neck cancers, with positron emission tomography fused with computerised tomography (PET-CT) in particular, contributing to the detection of incidental, usually subclinical thyroid tumours in their early stages [2, 3]. However, it may be incorrect to hastily interpret this recent surge as representative for all categories of thyroid cancer, simply because individual subtypes do not have the same phenotypic and pathological properties. For example, although the incidence of papillary thyroid cancer is thought to be rising [4], the global incidence of anaplastic thyroid cancer (ATC) over the last 30 years has remained stable with no significant upward or downward trend [5].

It is difficult to appraise data in the United Kingdom as there are limited national or regional studies that categorically explore the current annual incidence of ATC. Nonetheless, extrapolated findings from the 2021 British Association of Endocrine and Thyroid Surgeons (BAETS) sixth national audit report [6], combined with UK population data [7], place the annual incidence of ATC in the UK at 1.7 per 10,000,000 [6, 7].

The objective of this study was to explore the incidence of ATC in the North Yorkshire region between 2010 and 2022. Our results demonstrated a marked rise in the number of ATC cases within the 12-year period necessitating our investigation into its demographics and geographical distribution within North Yorkshire. We also intended to compare our findings with those of other regions in the country, but this proved challenging due to the paucity of data. We hope this paper serves to increase awareness throughout the UK to encourage the evaluation of local incidence trends of ATC. This could help to shed light on possible aetiologies if increased incidence is observed elsewhere in the UK, and appropriate measures could be instituted if modifiable risk factors are identified.

## Materials and methods

The study was conducted in York Teaching Hospital, which is the only tertiary centre for thyroid and parathyroid cases in North Yorkshire. North Yorkshire is one of the largest regions in the UK, comprising York, Harrogate, Scarborough, Selby, and Bridlington, amongst other towns, spanning across an area of 6430 square kilometres and having a population of about 1.1 million as of 2020 [8]. North Yorkshire is not known to have any nuclear power plant stations. The closest to the region is in Hartlepool, a small town in County Durham, which is at least 50 miles away.

This was a cross-sectional retrospective tertiary centre study. Inclusion criteria were records of patients with a diagnosis of ATC retrieved from the hospital's electronic core-patient database (ECPD) between 2010 and 2022 who were initially seen and evaluated in facilities within the North Yorkshire region. Exclusion criteria were records of patients with ATC retrieved from the hospital's ECPD who had been initially seen and evaluated in other facilities outside the North Yorkshire region. As this was a prevalence study rather than a hypothesis testing, there was no statistical test involved. The sampling technique involved the selection of all patients with ATC between 2010 and 2022 who had been initially seen and evaluated in facilities inside the North Yorkshire region.

Over the 12-year period, only 20 patient records matched the inclusion criteria, and they were retrieved and reviewed. Obtained data included age, gender, address, final histopathological diagnosis, and age at death. We determined North Yorkshire's ATC incidence rate by comparing the number of cases of ATC against the total population of the catchment area (North Yorkshire), which is estimated at 1.1 million [8], and also calculated its age-standardised incidence rate. The geographical distribution of the cases was represented by plotting each patient’s postcode on a 3D map using Microsoft Excel (Microsoft Corp., Redmond, WA) in an anonymised fashion to ensure patient confidentiality.

Since this was a retrospective study of patient case records with no alteration in interventions received by the patients nor involvement in clinical trials, approval with the exemption of patient consent was granted by the ethics committee of York Teaching Hospital. 

## Results

A total of 20 patients had a new diagnosis of anaplastic thyroid cancer between 2010 and 2022 in the North Yorkshire region, with 25% of them diagnosed in the last year. The mean age was 72.5 years. As the age range was 50 to 87 years, we calculated the median age and found it to be 76 years, thus reducing the effects of outliers. The male-to-female ratio was 1:1, while the five-year survival rate in this study was 5% (Table 1).

**Table 1:** Patient demographic table (North Yorkshire)

Variable	Frequency (number of patients)	Percentage (%)
Age at diagnosis (years)		
<50	0	0
50-70	8	40
>70	12	60
Mean age (years) 72.5; Median age (years) 76		
Gender		
Female	10	50
Male	10	50
Pre-existing thyroid disease		
Multinodular goitre	4	20
Thyroid nodule	2	10
Hashimoto thyroiditis	1	5
Goitre	1	5
None	12	60
Treatment received		
Tracheal stenting	2	10
Total thyroidectomy and adjuvant chemoradiation	2	10
Palliative radiotherapy	2	10
Best supportive care	14	70
Duration from diagnosis to death (days)		
Alive	6	30
<50	5	25
50-100	5	25
>100	4	20

**Figure 1:**
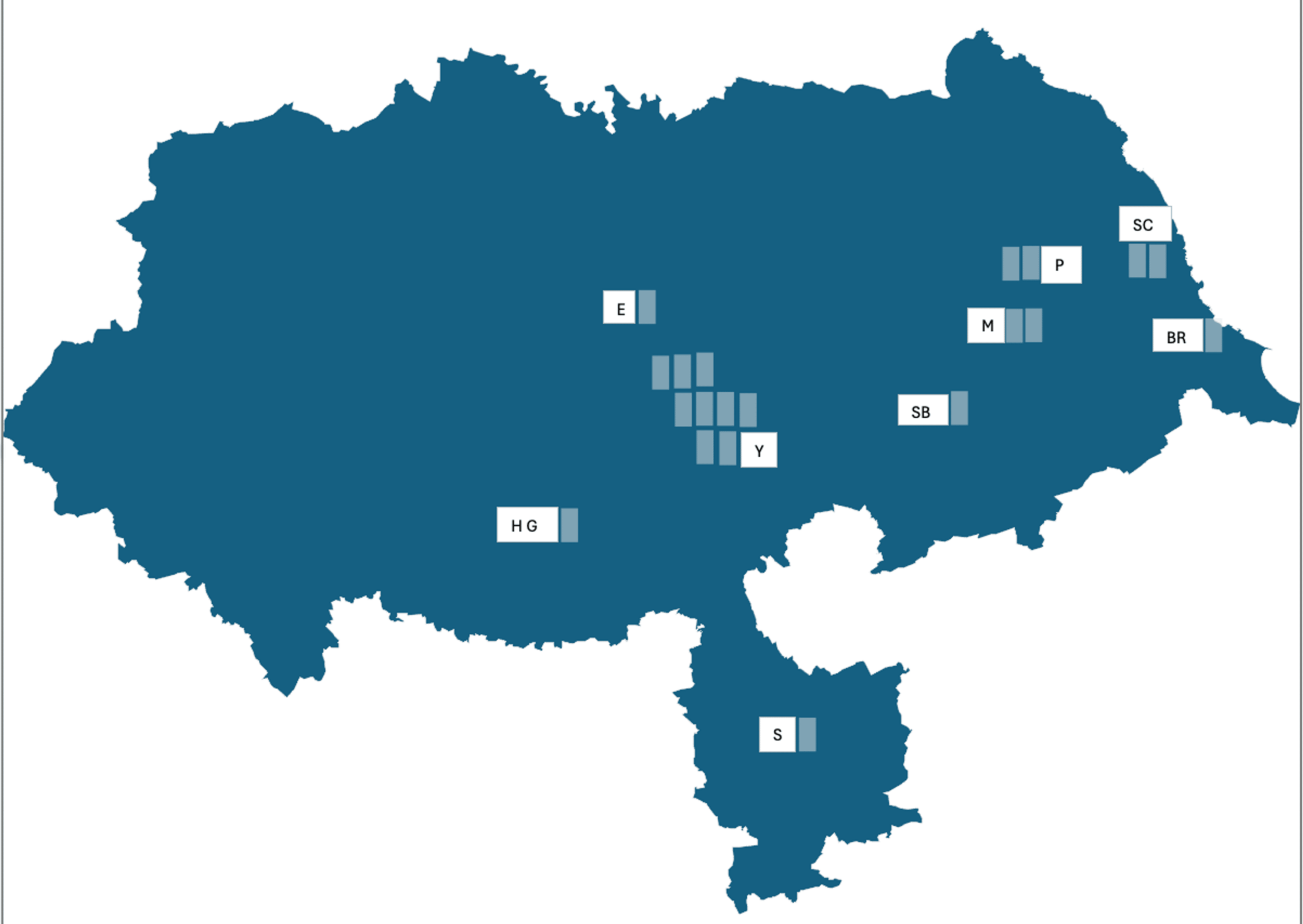
Geographical distribution indicating the incidence of anaplastic thyroid cancer cases in the North Yorkshire region of the United Kingdom between years 2010 and 2022 Y: York; HG: Harrogate; E: Easingwold; SC: Scarborough; SB: Stamford Bridge; S: Selby; P: Pickering; BR: Bridlington; M: Malton Adapted from https://www.plumplot.co.uk/North-Yorkshire-population.html [8]. Contains public sector information licensed under the Open Government Licence v3.0. This image has been created by the authors on Microsoft Excel (Microsoft Corp., Redmond, WA).

Within the data collection period, the extrapolated average annual incidence rate of ATC in North Yorkshire was 14 per 10,000,000 population, and there was a clustering of data in its centre with just a few cases in the periphery (Figure 1). The extrapolated annual national incidence rate of ATC in the United Kingdom was 1.7 per 10,000,000 population.

The ATC prevalence rate between 2010 and 2022 (12 years) in North Yorkshire = 20 cases divided by the North Yorkshire population of 1,158,816 = 172.6X10^7. The annual rate of incidence of ATC in North Yorkshire = 172.6 x 10^7 divided by 12 years = 14.4 x 10^7 = 14 per 10,000,000 population. The age-standardised incidence rate of ATC in the North Yorkshire region was 35 per 10,000,000 (Table 2). The ATC prevalence rate between 2016 and 2020 (five years) in the UK = 58 cases divided by the UK population of 67,081,234 = 8.6 x 10^7. The ATC annual incidence rate in the UK = 8.6 x 10^7 divided by five years = 1.7 x 10^7 = 1.7 per 10,000,000 population.

**Table 2:** Age-standardised incidence rate for ATC in North Yorkshire = 0.00004224 = (422.4 per 10,000,000 population) ÷ 12 years = 35.2 per 10,000,000 North Yorkshire population by age group source: https://www.plumplot.co.uk/North-Yorkshire-population.html [8]; Contains public sector information licensed under the Open Government Licence v3.0. ATC: anaplastic thyroid cancer

Age group (years)	Number ATC cases	North Yorkshire population by age group	Proportion of population (a)	Age-specific rate (b)	Age-standardised incidence rate (axb)
50-59	3	176,497	176,497/473,444 = 0.37279383	3/176,497 = 0.000017	0.00000634
60-69	5	135,925	135,925/473,444 = 0.28709837	5/135,925 = 0.00003678	0.00001056
70-79	6	109,994	109,994/473,444 = 0.23232737	6/109,994 = 0.00005455	0.00001267
80-89	6	51,028	51,028/473,444 = 0.10778043	6/51,028 = 0.00011758	0.00001267
Total	20	473,444	1	20/473,444 = 0.00004224	0.00004224

## Discussion

In this study, while a total of 20 patients were noted to have been diagnosed with ATC between the years 2010 and 2022, 25% of them had their ATC diagnosis in the last year of the data collection period (2022), and approximately 50% in the last five years (2018 to 2022). This depicts a dramatic rise in the number of cases over the study period, a rather unusual feature of anaplastic thyroid cancer, which is typically distinctive for its rarity. We are uncertain if this unprecedented finding is just an isolated event in North Yorkshire or if it is indeed the tip of the iceberg reflecting a recent nationwide surge in the incidence of ATC.

In our study, the overall mean and median ages were 72.5 years and 76 years, respectively. This is similar to a population-based study in the United States that arrived at a mean age of 70.5 years [9]. Although one of the patients in our study was well in her sixth year post-treatment for anaplastic thyroid cancer, the five-year survival rate in this study was 5%, which is similar to the 5%-7% commonly reported in different articles [10,11].

In Europe and other parts of the world, well-differentiated thyroid cancers are reported to occur more commonly in women with a female-to-male gender ratio of 3-4:1 [12]. The gender prevalence of poorly differentiated thyroid cancer has been generally variable, with some studies showing a slightly raised preponderance in females and others recording equal percentages. For example, in a study by Tavarelli and his colleagues in 2017 [12], a female predominance of 1.4:1 was observed; however, in a similar US study, equal prevalence was noted [13]. To the best of our knowledge and upon reviewing the literature, similar studies in the UK could not be found. Notably, our series echoed an equal gender ratio. Based on our data, it might be plausible to agree with another study that the more aggressive poorly differentiated thyroid cancers tend to breach the high female-to-male ratio characteristic seen with well-differentiated thyroid cancers [14].

Furthermore, comparing the annual regional incidence rate of ATC derived in this study, 14 per 10,000,000 entire North Yorkshire population (35 per 10,000,000 age-adjusted incidence rate), with the extrapolated UK national incidence rate of 1.7 per 10,000,000 population, the incidence rate in North Yorkshire was geometrically higher than the national figures. Whilst there was an obvious difference between the average national figures and the regional data in this study, the worldwide reports were consistent with the North Yorkshire figures. An international population-based study across 25 countries spread over five continents in 2021 reported the annual incidence rate of ATC to be about 20 per 10,000,000 population [1]. More specifically, in the US, the annual average incidence rate was 12 per 10,000,000 persons [9], and reports from the Netherlands showed an incidence rate of 18 per 10,000,000 people in 2020 [15]. Evidently, whilst our data in North Yorkshire correlates with international findings, the UK national data appears to differ with low values. This might raise a suspicion of underreporting in the BAETS national audit reports, the only available national reference in the UK to the best of our knowledge. However, this thought might not be entirely true as the annual incidence rate of well-differentiated thyroid cancer from the same BAETS audit report was 27 per 10,000,000 persons, which is consistent with our record of 14 per 10,000,000 of the entire North Yorkshire population (35 per 10,000,000 age-adjusted incidence rate). 

**Figure 2:**
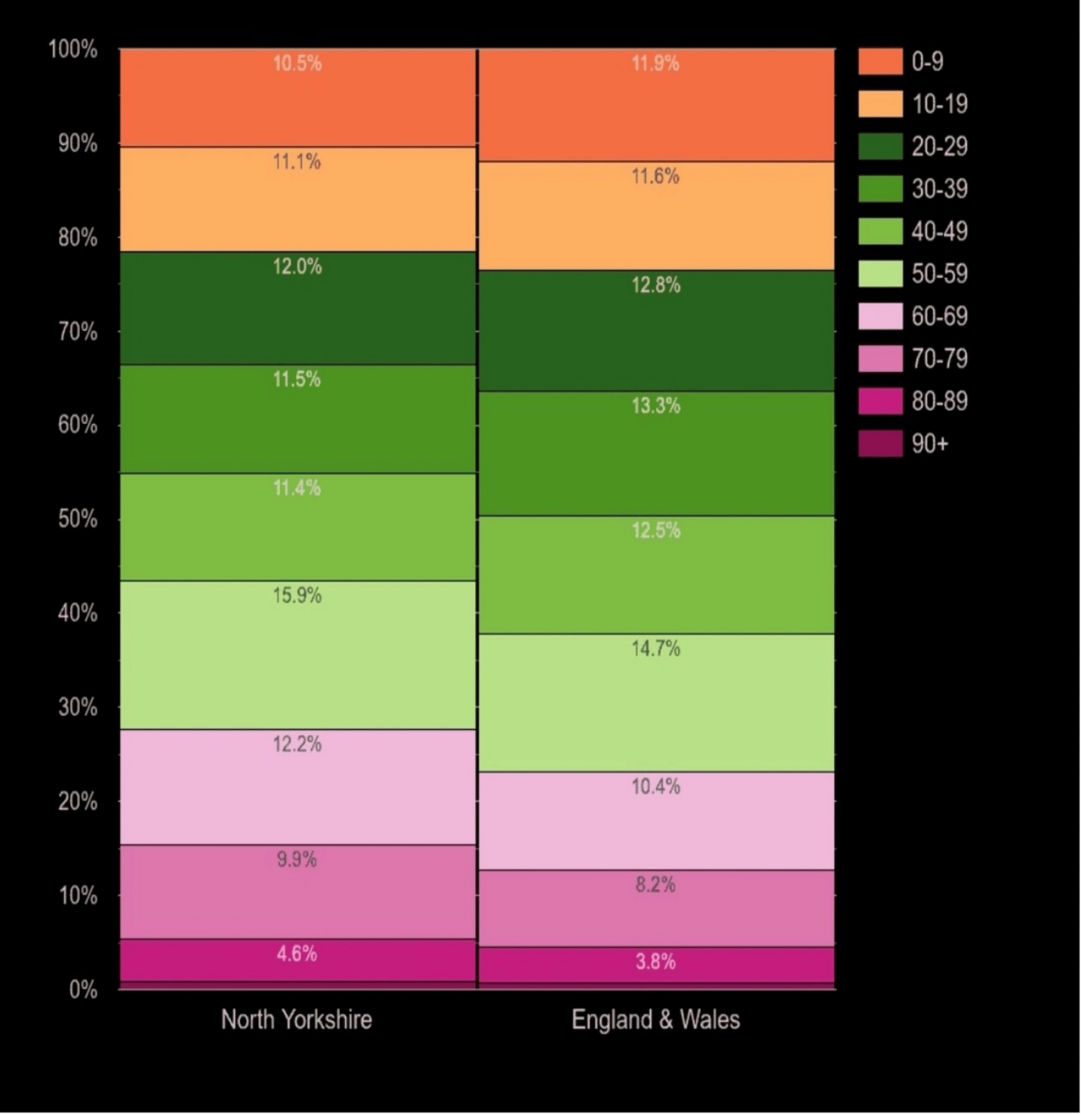
Distribution of population age groups in North Yorkshire compared to corresponding data in England and Wales, 2020 Reproduced from https://www.plumplot.co.uk/North-Yorkshire-population.html [8]. Contains public sector information licensed under the Open Government Licence v3.0.

Notably, North Yorkshire seems to have a slightly older population across all age groups compared to the rest of the UK [8] (Figure 2). Since ATC is usually more common in the elderly, the relative ageing population characteristic of North Yorkshire may account for the increased incidence observed in this study.

Study limitations

Although we found a disproportionately increased incidence rate of ATC in North Yorkshire, our study did not identify a reason for this. Whilst we explored the geographical distribution of cases for a possible cause, we found a clustering of data in the region's centre, which is likely explained by the relatively higher population density rather than by a specific, as a yet unidentified, environmental factor. As was systematically observed in the Derbyshire neck survey, where iodine deficiency was identified as a major cause of goitre endemicity in some UK districts many years ago [16], it would be prudent to investigate the cause of this markedly increased incidence rate in North Yorkshire, which could possibly include risk factors such as high-risk groups, inherited familial syndromes, and pre-existing thyroid pathologies. Whilst we intended to compare our North Yorkshire ATC age-adjusted incidence rate with its national equivalence, the paucity of data regarding age groups where ATC was diagnosed nationally in the UK from 2016 to 2020 rendered this difficult. 

## Conclusions

This study observed a disproportionate increase in the number of anaplastic thyroid cancer cases in the North Yorkshire region of the UK. Although the increased age of the North Yorkshire population relative to the UK may explain this, an analysis of the geographical distribution of cases did not help to elucidate a possible cause. It is therefore imperative to probe into the reason for this markedly increased incidence in the North Yorkshire region. Moreover, this is a subject for further studies on a national level, which could help to identify key environmental risk factors for anaplastic thyroid cancer. This may help to determine preventive measures to reduce the risk of developing ATC and potentially facilitate prompt disease detection with timely intervention in the hope of improving outcomes.
